# Mas Knockout Mice Present Altered Behavioral and Neuroendocrine Coping Responses to Chronic Unpredictable Stress

**DOI:** 10.1007/s12035-026-05747-6

**Published:** 2026-02-27

**Authors:** Sthéfanie C. A. Gonçalves, Andressa da Silveira Silva, Bruna Karen Oliveira Nogueira, Flávio A. G. Mourão, Marco Antônio Peliky Fontes, Michael Bader, Robson A. S. Santos, Maria José Campagnole-Santos, Lucas M. Kangussu

**Affiliations:** 1 National Institute of Science and Technology in Nanobiopharmaceutics (INCT-Nanobiofar), Belo Horizonte, Brazil; 2 Department of Physiology and Biophysics, Biological Sciences Institute, Universidade Federal de Minas Gerais (UFMG), Belo Horizonte, Brazil; 3 Department of Morphology, Biological Sciences Institute, Universidade Federal de Minas Gerais (UFMG), Belo Horizonte, Brazil; 4 Graduate Program in Neuroscience, Universidade Federal de Minas Gerais (UFMG), Belo Horizonte, Brazil; 5 Max-Delbrück Center for Molecular Medicine (MDC), Berlin, Germany; 6 German Center for Cardiovascular Research (DZHK), Partner Site Berlin, Berlin, Germany; 7 Institute for Biology, University of Lübeck, Lübeck, Germany; 8 Charité University Medicine Berlin, Berlin, Germany

**Keywords:** Chronic unpredictable stress, Renin-angiotensin system, Mas receptor, Anxiety-and depression-like behavior

## Abstract

Stress is defined as a disruption of homeostasis that elicits adaptive responses aimed at restoring physiological balance. However, when stress becomes chronic or overwhelming, maladaptive changes may occur, contributing to endocrine, behavioral, and neuropsychiatric dysfunctions. Beyond the classical neuroendocrine axes, such as the sympatho-adrenomedullary and hypothalamic–pituitary–adrenal (HPA) axes, the renin-angiotensin system has also being implicated in stress modulation. Previous studies have shown that angiotensin-(1–7), acting through its receptor Mas, exerts a modulatory effect on the stress response, attenuating anxiety- and depression-like behaviors induced by various stressors. Here we investigated the impact of genetic deletion of Mas on the consequences of chronic unpredictable stress (CUS) exposure. Over 21 consecutive days, mice were subjected to random stressors, after which endocrine, behavioral and neurochemical assessments were performed. Mas knockout (KO) mice exposed to CUS exhibited significantly elevated corticosterone and blood glucose levels compared to stressed wild-type mice. In behavioral tests, stressed Mas KO mice displayed the highest immobility times in the forced swimming test, indicating enhanced depressive-like behavior. Anxiety-like behavior was also heightened in Mas KO mice, as evidenced by a significant reduction in the percentage of time spent in the open arms of the elevated plus maze test. Neurochemical analysis revealed a marked reduction in brain-derived neurotrophic factor (BDNF) levels in key brain regions of stressed Mas KO animals. Together, these findings suggest that Mas plays a critical role in the neurobiology of stress, since its absence exacerbates HPA axis hyperactivity, depression- and anxiety-like behaviors, as well as BDNF reduction. Overall, these results highlight the potential neuroprotective role of Mas in stress-related disorders.

## Introduction

Stress is defined as a state that threatens homeostasis, thereby activating adaptive physiological mechanisms at restoring body balance [1]. This response includes increases in heart rate and blood glucose levels, the release of glucocorticoids (such as corticosterone in rodents) and catecholamines, as well as activation of the hypothalamic–pituitary–adrenal (HPA) and sympatho-adrenomedullary (SAM) axes. Together, these processes mobilize energy and prepare the organism to respond to environmental challenges [2, 3]. Stress can be categorized as acute, representing a rapid and transient response, or chronic, characterized by prolonged exposure to stressors that induce persistent physiological and behavioral changes [4, 5].

The renin-angiotensin system (RAS) is a key player in the neurobiology of stress, working alongside the canonical neurohumoral pathways. Although SAM and HPA axes are the primary mediators of the stress response, the RAS interacts with these systems to amplify or modulate their effects, thereby influencing our cardiovascular, endocrine, and behavioral responses to stress [6–9]. Within the RAS, angiotensin II (Ang II) acts as a stress-related hormone and contributes to cardiovascular and neurological dysfunctions under chronic conditions [6, 7, 10, 11]. In contrast, the counter-regulatory axis formed by angiotensin-(1–7) [Ang-(1–7)] acting though its receptor Mas exerts a modulatory effect on the stress response, attenuating anxiety- and depression-like behaviors induced by various stressors [8, 9, 12, 13].

The imbalance between Ang II and Ang-(1–7) has been implicated in the neurobiological and microvascular adverse effects of chronic stress [14–16]. Supporting this functional role, Mas receptor knockout (KO) in murine models is associated with cardiovascular impairments, including hypertension and dysfunctions in cardiovascular reflexes [17, 18], alterations in glucose and lipid metabolism [19], increased sympathetic activity [20], and impaired fear memory, suggesting its relevance as an animal model for the study of post-traumatic stress disorder (PTSD) [21]. In our previous studies, Mas KO mice also exhibited depression-like behavior accompanied by reduced BDNF levels [22]. BDNF plays a critical role in neurogenesis and neuronal maturation, and its reduction has been associated with depressive disorders [23–25].

Although the classical RAS axis is well established as a key contributor to chronic stress response, the role of the counter-regulatory ACE2/Ang-(1–7)/Mas receptor axis remains underexplored. Walther et al*.* (1998) reported anxiety-like behaviors in Mas KO mice [26], whereas Becari et al*.* (2022) demonstrated depression-like behavior in this lineage [22]. However, comprehensive studies on the effects of Mas deficiency on the neurobiology of CUS are still lacking. Given the potential role of Mas in stress regulation, we used the Mas KO mice to investigate the effects of Mas gene deletion on the endocrine, behavioral, and neurochemical outcomes following CUS exposure, testing the hypothesis that genetic deletion of Mas enhances stress-related disorders.

## Material and Methods

### Animals

**Fig. 1:**
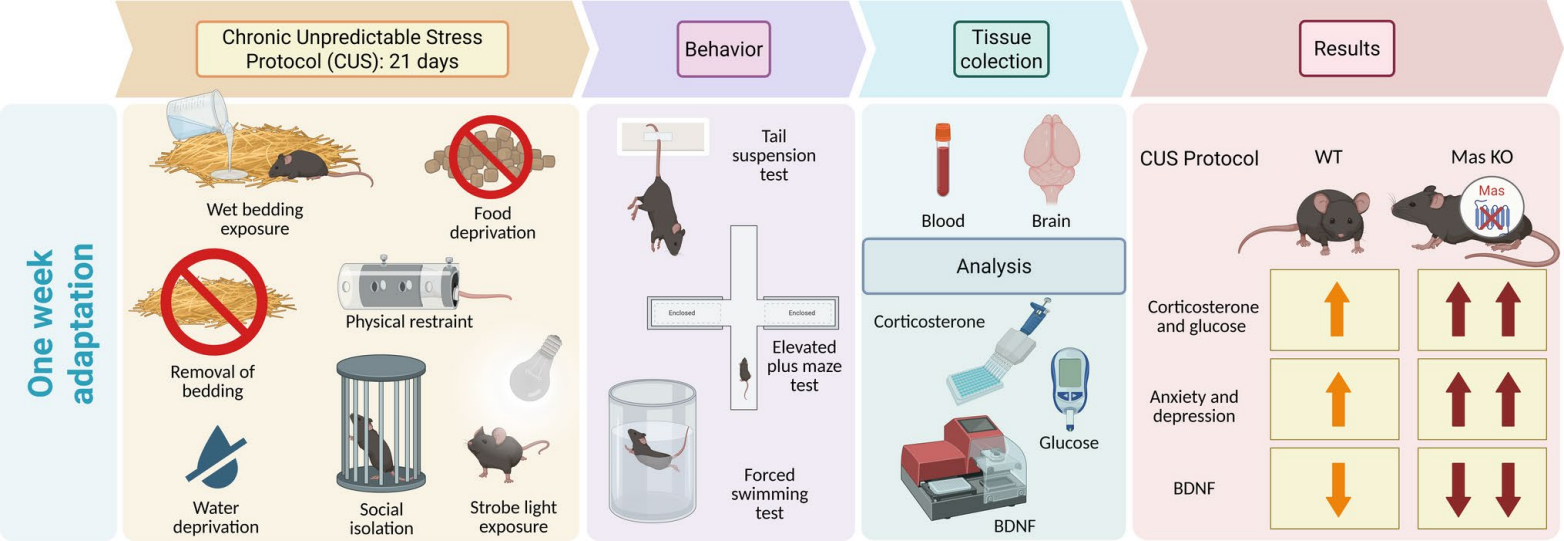
Timeline of the experimental protocol used to induce chronic unpredictable stress (CUS), followed by behavioral tests assessing depression-like and anxiety-like behaviors, as well as molecular analyses, including plasma corticosterone, blood glucose levels, and BDNF expression in the PFC, hippocampus, and hypothalamus (created with Biorender.com)

Adult male Mas KO (Mas KO) [26] mice (8–12 weeks of age) and their wild-type C57BL/6 J (WT) controls were used in this study. Animals were housed under controlled environmental conditions, with a temperature of 21–23 °C, relative humidity of 50–60%, and a 12 h light/dark cycle. All behavioral experiments were performed during the light phase, between 8.00 and 11.30 a.m. Mice were obtained from the Transgenic Animal Facility of the Hypertension Laboratory at the Institute of Biological Sciences, Federal University of Minas Gerais (ICB/UFMG), and were housed in the animal facility of the Department of Morphology at the same institute until the end of the study. Mice were kept in groups of 4–5 animals per polypropylene cage with food and water available ad libitum. All experiments were approved by the Institutional Ethics Committee on the Use of Animals at the Universidade Federal de Minas Gerais (CEUA/UFMG) (n^o^. 223/2020). All procedures were conducted in accordance with the guidelines of the Brazilian National Council for Control of Animal Experimentation (CONCEA-BRAZIL) and complied with the ARRIVE guidelines. The timeline of the experimental protocol is illustrated in Fig. 1.

### Chronic Unpredictable Stress (CUS)

The CUS protocol, originally described by Willner et al. (1987, 1992) [27, 28], was adapted in the present study to induce persistent stress-related behavioral alterations in mice. This model is based on the daily and random exposure to a variety of physical, social, and environmental stressors, thereby preventing habituation to the stimuli—a limitation commonly observed in other chronic stress paradigms [29, 30]. For 21 consecutive days, animals were exposed each day to a single stressor randomly selected from the following: water deprivation for 24 h, food deprivation for 24 h, wet bedding exposure for 6 h, strobe light exposure for 2 h, social isolation for 6 h, physical restraint for 1 h, bedding removal for 24 h. Stressors were preferentially applied during the light phase of the circadian cycle (between 8 a.m. and 12 p.m.) to ensure chronobiological consistency and minimize variability related to circadian hormonal fluctuations. Control animals were handled daily at the same time and in the same environment, but were not exposed to stressors, thereby controlling for handling effects without inducing stress.

### Plasma Corticosterone Levels

Plasma corticosterone levels were quantified using a mouse corticosterone (CORT) ELISA kit (#E-EL-0161)—Elabsience, according to the manufacturer’s instructions. For plasma collection, animals were anesthetized, and blood samples were obtained by cardiac puncture in the morning hours (between 8 a.m. and 11 a.m. to minimize circadian variability in corticosterone levels) on the day following completion of the CUS protocol. Blood samples were immediately transferred to tubes containing EDTA and centrifuged at 2000 rpm for 15 min at 4 °C. The resulting plasma was stored at –80 °C until analysis. All samples were analyzed in duplicate, and the absorbance was measured at 450 nm using a microplate reader (MULTISKAN EX, Thermo scientific). Corticosterone concentrations were calculated from a standard curve and the results were expressed as ng/ml.

### Blood Glucose

Fasting glucose levels were measured on the day following completion of the CUS protocol by collecting blood from a puncture at the tip of the mouse tail. Glucose concentrations were determined using reagent strips and a One Touch Ultra Plus Flex glucometer (Johnson & Johnson®), and the mean of the readings were expressed as mg/dL of blood.

### Behavioral Tests

Animals were acclimated to the experimental room for 2 h before the initiation.

of behavioral testing. All protocols were conducted during the morning, between 8:00 am and 12:00 am on the day following completion of the CUS protocol (day 22 after the start of the experimental assay). To avoid carry-over effects, animals were only used for one behavioral paradigm.

#### Tail Suspension Test (TST)

The TST was conducted according to previous studies from research group [22]. Briefly, mice were individually suspended by the tail using adhesive tape attached to a metal rod positioned 50 cm above the surface and allowed to hang upside down for 6 min. The immobility time, defined as the absence of any body movements except the related to respiration, was quantified in seconds by an experimenter blinded to the mice genotype. After the test, adhesive tape was gently removed, and the mice were returned in their home cages.

#### Forced Swimming Test (FST)

The FST was performed as previously described [22]. Experimentally naive Mas KO and WT mice were subjected to a single 6-min FST session, during which immobility time was measured during the last 4 min. The FST was performed in a glass cylinder (29 cm height × 18 cm diameter) filled with water to a depth of 20 cm at 25 °C. The sessions were video-recorded, and the total duration of immobility, defined as the absence of active movements except for those necessary to maintain flotation, was scored manually by an observer blinded to both genotype and treatment conditions (double-blind). Moreover, latency to first immobility episode was also measure, defined as the time to the first immobility bout lasting at least 2 s, starting immediately after the animal was placed in the cylinder [31, 32]. The water was changed after each trial to avoid the influence of alarm substances.

#### Elevated Plus Maze Test (EPM)

Experimentally naive Mas KO and WT were subjected to the EPM test for 5 min. The percentage of time spent in the open arms, the percentage of open-arm entries, and the number of closed-arm entries were analyzed using AnyMaze software (Stoelting, USA). The EPM apparatus was made of wood and consisted of two opposite open arms (30 cm × 6 cm) and two enclosed arms (30 cm × 6 cm × 5 cm), elevated 50 cm above the floor. After each animal section, the apparatus was cleaned with 10% ethanol.

### BDNF Levels

BDNF levels were quantified in the hypothalamus, prefrontal cortex, and hippocampus using a commercial ELISA kit (MyBioSource—MBS2700738), following the manufacturer’s instructions. Animals were euthanized, and the brain regions from both hemispheres were collected and homogenized using an Ultra-Turrax (T10 basic – IKA) in an extraction buffer (100 mg tissue/mL) containing 0.4 M NaCl, 0.05% Tween 20, 0.5% BSA, 0.1 mM phenyl methyl sulphonyl fluoride, 0.1 mM benzethonium chloride, 10 mM EDTA, and 20 KIU aprotinin. Homogenates were centrifuged at 13,000 × *g* fo 10 min at 4 °C, and the supernatants were collected for analysis. BDNF concentrations of BDNF were determined in duplicates and results were expressed as picograms per 100 mg of tissue.

### Statistical Analysis

Statistical analyses were performed using GraphPad Prism software (version 8.00, GraphPad Software, Inc.). Data normality was confirmed using the Kolmogorov–Smirnov test (*p* > 0.05). Group comparisons were conducted using two-way analysis of variance (ANOVA), followed by Tukey’s post hoc test when appropriate. Effect sizes for ANOVA were estimated using partial eta squared (ηp^2^), whereas effect sizes for pairwise comparisons were calculated using Cohen’s *d*. Data are presented as mean ± SEM, and differences were considered statistically significant at *p* < 0.05.

## Results

### Mas KO Mice Show Altered Plasma Corticosterone and Glucose Responses to CUS

Stress-related glucocorticoids are central mediators of responses to environmental and psychological stress. Stress-induced activation of the hypothalamic–pituitary–adrenal (HPA) axis promotes corticosterone release, which regulates glucose homeostasis by enhancing gluconeogenesis, limiting peripheral glucose uptake, and mobilizing energy substrates [33]. Given the potential role of Mas in stress regulation, we assessed corticosterone levels and capillary blood glucose in response to the CUS protocol.

**Fig. 2:**
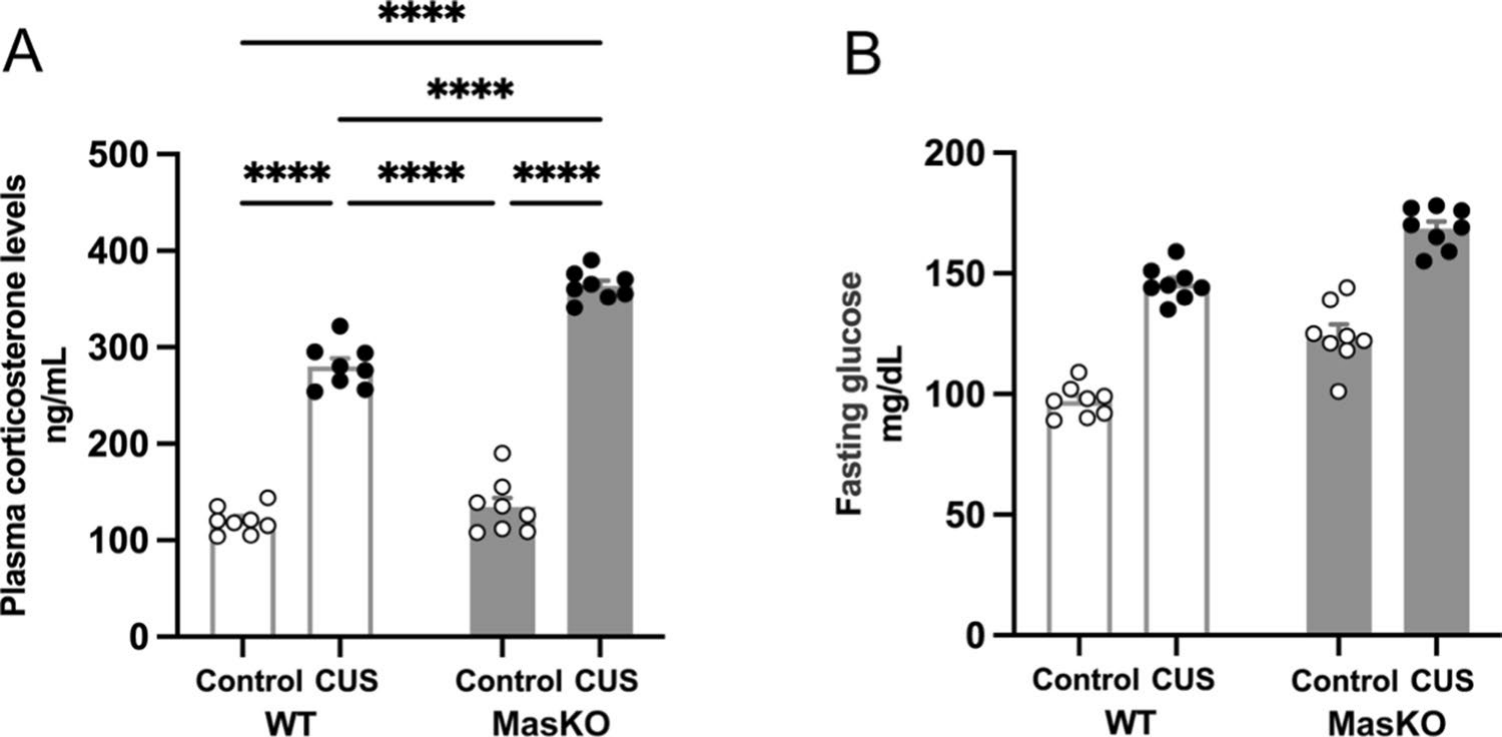
Analysis of plasma corticosterone levels (**A**) and capillary blood glucose levels during the fasting period (**B**) in wild-type (WT) and Mas knockout (Mas KO) mice exposed to chronic unpredictable stress (CUS) or control conditions. Data are expressed as mean ± SEM (*n* = 8 per group). Statistical analyses were performed using two-way ANOVA followed by Tukey’s post hoc test. ****p < 0.0001

According to the results, plasma corticosterone levels were markedly influenced by stress exposure and genotype. Statistical analysis revealed significant main effects of CUS protocol (F_(1,28)_ = 703.6, *p* < 0.0001, ηp^2^ = 0.96) and genotype (F_(1,28)_ = 44.01, *p* < 0.0001, ηp^2^ = 0.61), as well as a significant CUS protocol × genotype interaction (F_(1,28)_ = 22.34, *p* < 0.0001, ηp^2^ = 0.44). Tukey post hoc test showed that CUS produced an elevation in corticosterone levels in both genotypes, with particularly large effect sizes relative to control conditions (*p* < 0.0001, Cohen’s d > 3). Under CUS, Mas KO mice exhibited higher corticosterone levels compared to WT mice (WT CUS: 280 ± 8.1; Mas KO CUS: 364 ± 5.4; *p* < 0.0001; Cohen’s d = 1.35), whereas corticosterone levels did not differ between genotypes under control conditions (WT: 120 ± 4.8; Mas KO: 134 ± 9.9). The significant interaction indicates an increased stress-induced corticosterone response in Mas KO mice, supporting a role for Mas signaling in modulating HPA axis reactivity to chronic stress (Fig. 2A).

Furthermore, blood glucose levels were also significantly influenced by stress exposure and genotype with a main effect of CUS protocol (F_(1,28)_ = 203.6, *p* < 0.0001, ηp^2^ = 0.88) and genotype (F_(1,28)_ = 59.00, p < 0.0001, ηp^2^ = 0.68). In contrast, the CUS protocol × genotype interaction was not significant (F_(1,28)_ = 0.45, *p* = 0.508, ηp^2^ = 0.02), indicating that the effects of CUS on glucose levels were comparable across genotypes. These findings indicate that stress exposure and genotype independently affect glucose homeostasis. Both WT and Mas KO mice showed elevated glucose levels following CUS. However, Mas KO mice exhibited higher mean glucose levels than WT mice under both control and stress conditions (Fig. 2B).

### Mas KO Mice are more Susceptible to Depression-Like Behaviors

The TST and FST are behavioral assays widely used to assess depression-like behaviors in rodents. In both assays, increased immobility is interpreted as a behavioral index of depression-like states [31].

**Fig. 3:**
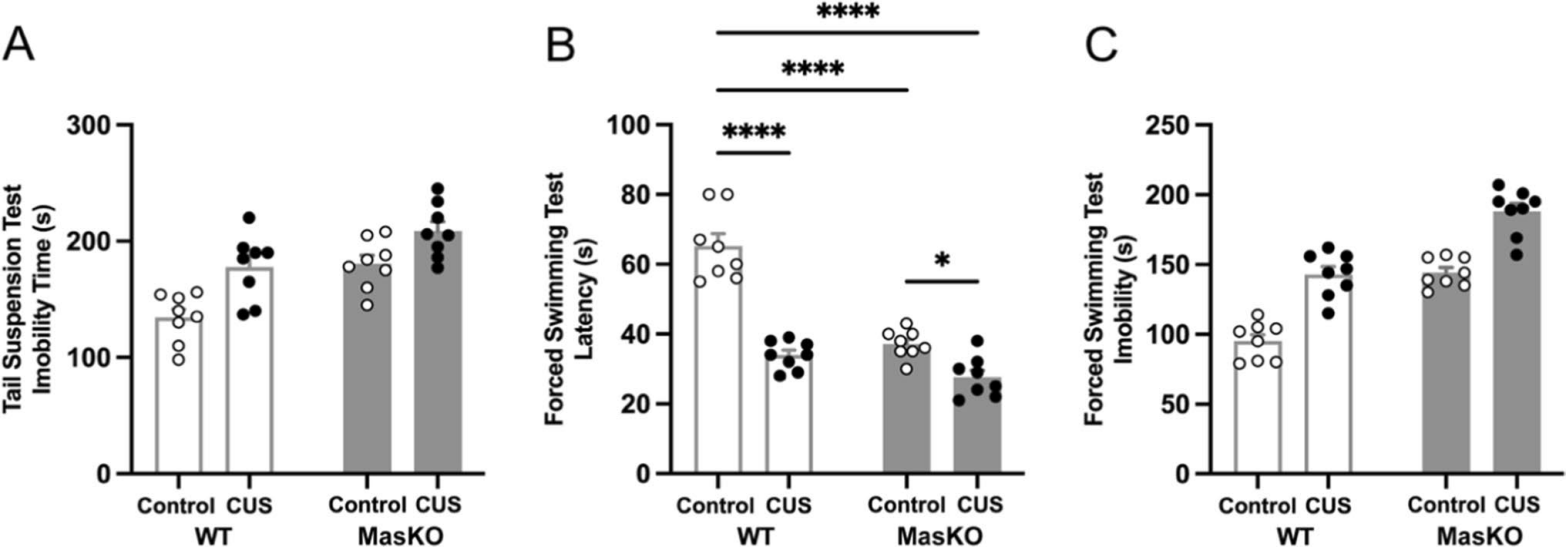
Depression-like behavior assessed by the Tail Suspension Test (**A**), latency to the first immobility episode (**B**), and total immobility time in the Forced Swim Test (**C**) in wild-type (WT) and Mas knockout (Mas KO) mice exposed to chronic unpredictable stress (CUS) or control conditions. Data are expressed as mean ± SEM (*n* = 8 per group). Statistical analyses were performed using two-way ANOVA followed by Tukey’s post hoc test. *p < 0.05, ****p < 0.0001

As supported by the analysis, immobility time in the TST was significantly influenced by stress exposure and genotype with a significant main effect of CUS protocol (F_(1,28)_ = 18.23, *p* = 0.0002, ηp^2^ = 0.39) and genotype (F_(1,28)_ = 21.14, *p* < 0.0001, ηp^2^ = 0.43), but the CUS protocol × genotype interaction was not significant (F_(1,28)_ = 0.83, *p* = 0.37, ηp^2^ = 0.03). The results indicate independent effects of stress exposure and genotype on immobility in the TST, with Mas KO mice exhibiting higher mean immobility time than WT mice across conditions (Fig. 3A).

In the FST, both latency to first immobility and total immobility time were significantly influenced by stress exposure and genotype. For latency to first immobility, there was a considerable main effect of CUS protocol (F_(1,28)_ = 79.72, *p* < 0.0001, ηp^2^ = 0.74) and genotype (F_(1,28)_ = 56.32, *p* < 0.0001, ηp^2^ = 0.67), as well as a significant CUS protocol × genotype interaction (F_(1,28)_ = 22.71, *p* < 0.0001, ηp^2^ = 0.45). Tukey post hoc test showed that, under control conditions, Mas KO mice exhibited significantly shorter latencies to immobility than WT mice (WT: 65 ± 3.6, Mas KO: 37 ± 1.4, *p* < 0.0001, Cohen’s d = 1.05), in line with the hypothesis that Mas KO mice exhibit increased vulnerability to stress and related depression-like behavior. CUS exposure significantly reduced latency in WT mice (34 ± 1.4, *p* < 0.0001, Cohen’s d = 1.17) and in Mas KO mice (28 ± 2, *p* = 0.0309, Cohen’s d = 0.84) compared to the control groups. Comparisons between WT and Mas KO mice under CUS conditions did not reach significance (*p* = 0.236) (Fig. 3B). Furthermore, total immobility time showed significant main effects of CUS protocol (F_(1,28)_ = 81.61, *p* < 0.0001, ηp^2^ = 0.74) and genotype (F_(1,28)_ = 86.12, *p* < 0.0001, ηp^2^ = 0.75), with no significant CUS protocol × genotype interaction (F_(1,28)_ = 0.17, *p* = 0.69, ηp^2^ = 0.01). These results indicate that stress exposure and genotype independently increase overall immobility in the FST. Accordingly, Mas KO mice display higher immobility levels across conditions (Fig. 3C).

### Mas KO Mice are more Susceptible to Anxiety-Like Behaviors

The EPM is a classical behavioral assay to assess anxiety-like behavior in rodents. It is based on the natural conflict between exploration of open spaces and avoidance of elevated, exposed areas. Reduced exploration of the open arms is interpreted as increased anxiety-like behavior [34].

**Fig. 4:**
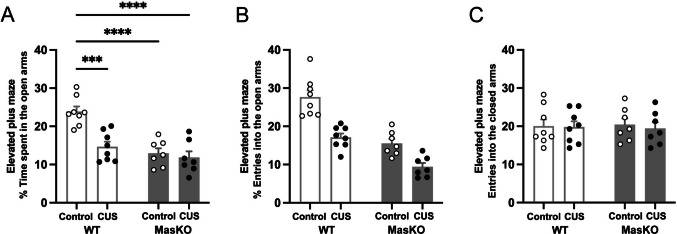
Anxiety-like behavior assessed by the elevated plus maze. Percentage of time spent in the open arms (**A**), percentage of entries into the open-arm entries (**B**), and number of entries into the closed arms (**C**) in wild-type (WT, n = 8) and Mas knockout (Mas KO, n = 7) mice exposed to chronic unpredictable stress (CUS) or control conditions. Data are expressed as mean ± SEM. Statistical analyses were performed using two-way ANOVA followed by Tukey’s post hoc test. ***p < 0.001, ****p < 0.0001

In line with the previous behavioral findings, the percentage of time spent in the open arms was significantly influenced by stress exposure and genotype. The analysis revealed significant main effects of CUS protocol (F_(1,26)_ = 13.56, *p* = 0.0011, ηp^2^ = 0.34) and genotype (F_(1,26)_ = 24.00, *p* < 0.0001, ηp^2^ = 0.48), as well as a significant Treatment × Group interaction (F_(1,26)_ = 8.40, *p* = 0.0075, ηp^2^ = 0.24). Under control conditions, Mas KO mice spent a significantly lower percentage of time in the open arms compared to WT mice (WT: 24 ± 1.3, Mas KO: 13 ± 1.3, *p* < 0.0001, Cohen’s d = 1.06), suggesting a heightened anxiety-like phenotype. CUS exposure significantly reduced open-arm exploration in WT mice (15 ± 1.3, *p* = 0.0003, Cohen’s d = 0.89), whereas Mas KO mice exhibited similarly low open-arm exploration under both control and stress conditions (12 ± 1.6). Together, these findings reveal genotype-dependent differences in anxiety-like behavior, with Mas KO mice displaying reduced open-arm exploration (Fig. 4A). For the percentage of entries into the open arms, robust main effects of CUS protocol (F_(1,26)_ = 38.82, *p* < 0.0001, ηp^2^ = 0.60) and genotype (F_(1,26)_ = 55.03, *p* < 0.0001, ηp^2^ = 0.68) were observed. CUS protocol × genotype interaction did not reach significance (F_(1,26)_ = 2.74, *p* = 0.11, ηp^2^ = 0.10) (Fig. 4B). No significant effects of stress exposure, genotype, or their interaction were observed for the number of closed arm entries (Fig. 4C).

### Mas KO Mice Exhibit Altered Stress-Induced BDNF Regulation Across the PFC, Hippocampus, and Hypothalamus

**Fig. 5:**
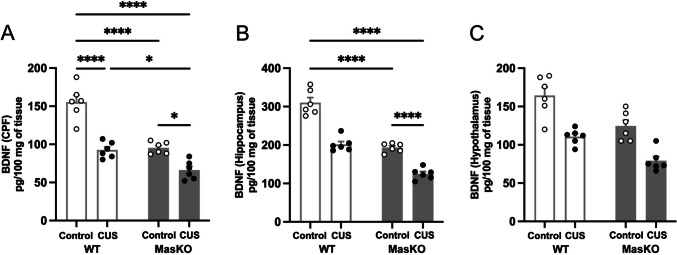
Quantification of BDNF levels in the prefrontal cortex (PFC) (**A**), hippocampus (**B**), and hypothalamus (**C**) in wild-type (WT) and Mas knockout (Mas KO) mice exposed to chronic unpredictable stress (CUS) or control conditions. Data are expressed as mean ± SEM (n = 6 per group). Statistical analyses were performed using two-way ANOVA followed by Tukey’s post hoc test. *p < 0.05, ****p < 0.0001

BDNF is a key regulator of synaptic plasticity and neuronal survival. Stress has been shown to alter BDNF expression in brain regions involved in emotional regulation and cognition [23]. PFC BDNF levels showed a robust main effect of the CUS protocol (F_(1,20)_ = 61.27, *p* < 0.0001, ηp^2^ = 0.75) and genotype (F_(1,20)_ = 54.36, *p* < 0.0001, ηp^2^ = 0.73), as well as a significant CUS protocol × genotype interaction (F_(1,20)_ = 8.15, *p* = 0.0098, ηp^2^ = 0.29). Tukey post hoc analyses showed that, under control conditions, Mas KO mice exhibited significantly lower BDNF levels than WT mice (WT: 155 ± 9.2, Mas KO: 95 ± 3.2, *p* < 0.0001, Cohen’s d = 1.21). CUS exposure reduced BDNF levels in WT mice (93 ± 4.3, *p* < 0.0001, Cohen’s d = 1.26) and produced a further reduction in Mas KO mice (66 ± 5, *p* = 0.0107, Cohen’s d = 1.50), with Mas KO mice displaying significantly lower BDNF levels than WT mice under stress conditions (*p* = 0.0217, Cohen’s d = 1.02). These findings indicate that Mas deletion is associated with a heightened vulnerability to stress-induced disruptions in cortical plasticity (Fig. 5A).

Hippocampal BDNF levels showed a pattern similar to that observed in the PFC showing a considerable main effect of CUS protocol (F_(1,20)_ = 105.3, *p* < 0.0001, ηp^2^ = 0.84) and genotype (F_(1,20)_ = 126.7, *p* < 0.0001, ηp^2^ = 0.86), along with a significant CUS protocol × genotype interaction (F_(1,20)_ = 5.50, *p* = 0.0294, ηp^2^ = 0.22). Tukey post hoc analyses also showed that, under control conditions, Mas KO mice exhibited significantly lower hippocampal BDNF levels than WT mice (WT: 310 ± 13, Mas KO: 193 ± 4.8, *p* < 0.0001, Cohen’s d = 1.58). CUS exposure reduced BDNF levels in WT mice (202 ± 7.6, *p* < 0.0001, Cohen’s d = 1.45) and produced a substantially reduction in Mas KO mice (125 ± 6, *p* < 0.0001, Cohen’s d = 2.36), with stressed Mas KO mice displaying significantly lower BDNF levels than stressed WT mice (*p* < 0.0001, Cohen’s d = 1.75) (Fig. 5B).

Finally, in the hypothalamus, BDNF levels analysis revealed a significant main effect of CUS protocol (F_(1,20)_ = 43.27, *p* < 0.0001, ηp^2^ = 0.68) and genotype (F_(1,20)_ = 22.46, *p* = 0.0001, ηp^2^ = 0.53). In contrast to the PFC and hippocampus, the CUS protocol × genotype interaction was not significant (F_(1,20)_ = 0.31, *p* = 0.586, ηp^2^ = 0.02), indicating that stress exposure and genotype independently influenced hypothalamic BDNF levels. These results suggest that in the hypothalamus BDNF levels are further reduced by stress exposure in both genotypes, despite already low means values in Mas KO mice (Fig. 5C).

## Discussion

This is the first study describing that genetic deletion of the Ang-(1–7) receptor Mas critically modulates the neurobiological and behavioral consequences of CUS. The absence of this receptor exacerbated canonical stress-associated alterations, including marked hyperactivity of the HPA axis, as reflected by elevated plasma corticosterone and fasting glucose levels. Moreover, anxiety- and depression-like behaviors were observed, accompanied by a significant reduction in BDNF levels in the prefrontal cortex, hippocampus, and hypothalamus, key brain regions involved in emotional processing and stress regulation, suggesting impaired stress adaptation. These findings reinforce the pivotal role of Mas and its relevance to the neurobiology of stress-related disorders.

Hyperactivation of the HPA axis, as evidenced by elevated plasma corticosterone levels, was observed in both genotypes exposed to CUS, as expected and consistent with previous reports [35]. Uniquely, our data demonstrate that Mas receptor deletion further amplified this neuroendocrine response, indicating a potential modulatory role of Mas in HPA axis regulation. This hyperactivation may be directly implicated in the observed anxiety- and depression-like behaviors, as excessive glucocorticoid exposure negatively affects limbic structures such as the hippocampus and amygdala, which are critically involved in emotional regulation [36–38].

All components of RAS are expressed in the brain, where angiotensin peptides have long been associated with cardiovascular regulation [39, 40]. Beyond these classical cardiovascular roles, early studies localized Mas receptor expression primarily to the hippocampal formation, limbic structure critically involved in cognitive and emotional processing [41]. This anatomical distribution initially guided phenotypic analyses of Mas-deficient mice toward affective behavioral alterations [26]. Subsequent investigations expanded this view by demonstrating Mas expression in multiple brain regions involved in autonomic and neuroendocrine regulation, indicating a broader functional role for central Mas signaling [42].

In line with these observations, the central Ang-(1–7)/Mas axis has emerged as an important modulator of emotional stress and anxiety-related behaviors [43]. Accordingly, central administration of Ang-(1–7) attenuates stress-induced tachycardia, suggesting that Ang-(1–7)/Mas signaling modulates stress responses through central anxiolytic mechanisms and/or interactions with β-adrenergic cardiac chronotropic pathways [44]. Furthermore, the lack of Mas signaling may favors the predominance of pro-stress pathways mediated by angiotensin II and the AT_1_ receptor activation [9, 45]. Additionally, Mas activation has been associated with anxiolytic and antidepressant-like effects in rodent models [46–50], suggesting that its absence reduced protective modulation against stress.

In our study, behavioral assays designed to evaluate depressive-like phenotypes confirmed the effectiveness of the CUS protocol in inducing behavioral alterations consistent with psychiatric disorders, as reflected by increased immobility in both the FST and TST. Notably, Mas KO mice exhibited a significant exacerbation of these depressive-like behaviors, caractherized by reduced latency to immobility in FST. These findings are align with previous evidence from our group, demonstrating enhaced depressive-like behavior in Mas KO male mice in both FST and TST [22]. A possible explanation for why the CUS x genotype interaction was detected only in latency to immobility is that this parameter is often considered more sensitive than total immobility time in the FST [51].

Importantly, the enhanced anxiety-like behavior in Mas KO mice subjected to CUS, as evidenced by reduced the percentage of time spent in the open arms of the elevated plus maze, suggests that the absence of Mas increases susceptibility to the anxiogenic effects of CUS, resulting in more pronounced behavioral alterations compared to the control animals exposed to the same stress protocol. Our results are in accordance with previous findings where Mas-deficient animals display increased anxiety-like behavior in the elevated plus maze [26]. Conversely, increased endogenous Ang-(1–7) levels in transgenic rats reduce renal sympathetic outflow, attenuate cardiovascular responses to emotional stress, and decreased anxiety-like behavior, effects that are abolished by central Mas antagonism A779 [49]. Similary, Wang et al. (2016) showed that the anxiolytic phenotype associated with ACE2 overexpression is dependent on Mas signaling, as this effect is reversed by central administration of the A779 [47], additionally Ang-(1–7) mediates anxiolytic effects through central Mas-dependent mechanisms involving NMDAR–nNOS–NO signaling and serotonergic pathways [13].

We also analysed BDNF levels and found a significant reduction in the prefrontal cortex, hippocampus, and hypothalamus of Mas KO mice, an effect that was exacerbated by CUS exposure. BDNF is essencial for neuronal development, survival, and synaptic plasticity and reduced BDNF signaling has been strongly associated in the pathophysiology of depression due to its negative effects on neuroplastic processes, including neurogenesis, synaptogenesis, and synaptic plasticity [23, 52–54]. Likewise, chronic stress substantially decreases BDNF expression [55], with evidence indicating that stress reduces BDNF transcript levels, whereas elevated corticosterone selectively impairs BDNF protein expression [23, 56]. In line with a causal role for BDNF in stress vulnerability, mice with microglia-specific BDNF deficiency display increased susceptibility to the behavioral and cognitive consequences of stress [57]. Notably, Becari et al., (2022) demonstrated that Mas deletion alone is sufficient to reduce BDNF levels even in the absence of stress [22]. Extending this findings, our data show that CUS further potentiates this reduction in Mas KO mice, suggesting that Mas signaling functions as a critical neurobiological resilience mechanism that buffers stress-induced impairments in BDNF-dependent plasticity. Mechanistically, the Ang-(1–7))/Mas receptor axis has been shown to positively regulate BDNF availability by limiting oxidative stress, and functionally opposing the deleterious actions of Ang II. These effects are particularly evident in models of neurodegeneration and cognitive dysfunction, where Mas signaling engages pro-plasticity intracellular cascades, including PI3K/Akt and CREB activation, ultimately enhancing BDNF expression and TrkB-dependent neuroprotective signaling [58].

It is important to highlight that reductions in BDNF exert distinct functional consequences depending on the brain region affected, even though the underlying mechanisms are closely related. In the hippocampus, decreased BDNF is primarily associated with impaired synaptic plasticity [59], reduced adult neurogenesis, and deficits in learning and memory, as well as increased vulnerability to depressive-like behaviors [60]. In the prefrontal cortex, reduced BDNF compromises top-down regulation of emotional responses, impaired cognitive flexibility, and maladaptive stress coping [61]. In contrast, decreased hypothalamic BDNF predominantly reflects disruptions in neuroendocrine and autonomic regulation, leading to altered HPA axis activity and exaggerated stress hormone responses [62]. Collectively, these region-specific alterations suggest that BDNF loss contributes to stress-related pathology through complementary mechanisms affecting cognitive processing, emotional regulation, and physiological homeostasis.

We acknowledge limitations of the present study. First, the exclusive use of male mice limits the generalizability of the results, as sex-specific hormonal fluctuations in females may substantially modulate stress responses and, consequently, the effects of Mas deletion. Second, the absence of experimental groups treated with selective Mas agonists or antagonists limits a direct pharmacological assessment of Mas function. In addiction, complementary molecular analyses, such as gene expression profiling and the assessement of inflammatory marker evaluation, were not performed, and could yield further insight into the mechanisms the observed behavioral and neubiological effects. Accordingly, future studies should explore the therapeutic potential of selective Mas agonists and investigate the molecular and epigenetic mechanisms involved in its action.

Taken together, our findings demonstrate that the genetic deletion of the Mas exacerbates hallmark features of chronic stress, including HPA axis hyperactivation, anxiety- and depression-like behaviors, and reductions in BDNF levels within key brain regions involved in emotional regulation. These results identify Mas as a critical modulator of neuroendocrine, behavioral, and neurotrophic adaptations to chronic stress. Finally, our study broaden the understanding of the brain renin-angiotensin system and suggests that Mas signaling may represent a promising therapeutic target for treatment of stress-related disorders.

## Data Availability

All data supporting the findings of this study are available within the paper and its Supplementary Information.
